# Robotic versus laparoscopic distal pancreatectomy in obese patients

**DOI:** 10.1007/s00464-023-10361-x

**Published:** 2023-09-15

**Authors:** Fabio Ausania, Filippo Landi, John B. Martinie, Dionisios Vrochides, Matthew Walsh, Shanaz M. Hossain, Steven White, Viswakumar Prabakaran, Laleh G. Melstrom, Yuman Fong, Giovanni Butturini, Laura Bignotto, Valentina Valle, Yuntao Bing, Dianrong Xiu, Gregorio Di Franco, Francisco Sanchez-Bueno, Nicola de’Angelis, Alexis Laurent, Giuseppe Giuliani, Graziano Pernazza, Alessandro Esposito, Roberto Salvia, Francesca Bazzocchi, Ludovica Esposito, Andrea Pietrabissa, Luigi Pugliese, Riccardo Memeo, Ichiro Uyama, Yuichiro Uchida, José Rios, Andrea Coratti, Luca Morelli, Pier C. Giulianotti

**Affiliations:** 1 Department of HBP Surgery and Transplantation, General and Digestive Surgery, Hospital Clinic Barcelona, Institut d’Investigacions Biomèdiques August Pi i Sunyer (IDIBAPS), University of Barcelona (UB), C. Villarroel, 170, 08036 Barcelona, Spain; 2 Division of HPB Surgery, Department of Surgery, Carolinas Medical Center, Charlotte, NC USA; 3 HPB Surgery Department, Digestive Disease and Surgery Institute, Cleveland Clinic, Cleveland, OH USA; 4 Freeman Hospital, NHS, Newcastle-Upon-Tyne, UK; 5 Division of Surgical Oncology, Gastrointestinal Disease Team, City of Hope Medical Center, Duarte, CA USA; 6 Department of HBP Surgery, P. Pederzoli Hospital, Peschiera del Garda, Italy; 7 Division of General, Minimally Invasive and Robotic Surgery, Department of Surgery, University of Illinois at Chicago, Chicago, IL USA; 8 Department of General Surgery, Beijing Third Hospital, Beijing, China; 9 Division of Translational and New Technologies in Medicine and Surgery, General Surgery Department, University of Pisa, Pisa, Italy; 10 Department of HBP Surgery, Virgen de la Arrixaca Hospital, Murcia, Spain; 11 Department of Digestive, HBP Surgery and Liver Transplantation, Henri Mondor Hospital, APHP, Creteil, France; 12 Division of General and Minimally Invasive Surgery, Misericordia Hospital, Grosseto, Italy; 13 General and Robotic Surgery Department, San Giovanni Hospital, Rome, Italy; 14 HBP Surgery Department, Policlinico G.B. Rossi Hospital, Verona, Italy; 15 Department of HBP Surgery, IRCCS Casa Sollievo della Sofferenza Hospital, Foggia, Italy; 16 Department of HBP Surgery, Policlinico S. Matteo Hospital, Pavia, Italy; 17 Department of Surgery, Acquaviva delle Fonti Hospital, Bari, Italy; 18 Department of Surgery, Fujita Health University School of Medicine, Toyoake, Japan; 19 Department of Clinical Pharmacology, Hospital Clinic and Medical Statistics Core Facility, Institut d’Investigacions Biomèdiques August Pi i Sunyer (IDIBAPS), Biostatistics Unit, Faculty of Medicine, Universitat Autònoma de Barcelona (UAB), Barcelona, Spain; 20 Facultat de Medicina i Ciències de la Salut, Universitat de Barcelona (UB), Barcelona, Spain

**Keywords:** Left pancreatic resection, Laparoscopic distal pancreatectomy, Robotic distal pancreatectomy, Obesity, Outcomes

## Abstract

**Background:**

Although robotic distal pancreatectomy (RDP) has a lower conversion rate to open surgery and causes less blood loss than laparoscopic distal pancreatectomy (LDP), clear evidence on the impact of the surgical approach on morbidity is lacking. Prior studies have shown a higher rate of complications among obese patients undergoing pancreatectomy. The primary aim of this study is to compare short-term outcomes of RDP vs. LDP in patients with a BMI ≥ 30.

**Methods:**

In this multicenter study, all obese patients who underwent RDP or LDP for any indication between 2012 and 2022 at 18 international expert centers were included. The baseline characteristics underwent inverse probability treatment weighting to minimize allocation bias.

**Results:**

Of 446 patients, 219 (50.2%) patients underwent RDP. The median age was 60 years, the median BMI was 33 (31–36), and the preoperative diagnosis was ductal adenocarcinoma in 21% of cases. The conversion rate was 19.9%, the overall complication rate was 57.8%, and the 90-day mortality rate was 0.7% (3 patients). RDP was associated with a lower complication rate (OR 0.68, 95% CI 0.52–0.89; p = 0.005), less blood loss (150 vs. 200 ml; p < 0.001), fewer blood transfusion requirements (OR 0.28, 95% CI 0.15–0.50; p < 0.001) and a lower Comprehensive Complications Index (8.7 vs. 8.9, p < 0.001) than LPD. RPD had a lower conversion rate (OR 0.27, 95% CI 0.19–0.39; p < 0.001) and achieved better spleen preservation rate (OR 1.96, 95% CI 1.13–3.39; p = 0.016) than LPD.

**Conclusions:**

In obese patients, RDP is associated with a lower conversion rate, fewer complications and better short-term outcomes than LPD.

Minimally invasive distal pancreatectomy (MIDP) techniques, including robotic (RDP) and laparoscopic (LDP) approaches, are known to be safe options for selected patients undergoing left pancreatectomy [1, 2]. RDP has been increasingly performed, and previous authors have demonstrated that it is associated with a lower rate of conversion to open surgery and less blood loss than LDP [3, 4]. However, there is limited evidence that the robotic approach is superior to LPD in terms of postoperative complications [5]. In fact, recent retrospective studies have demonstrated that the major morbidity rate is comparable between RDP and LDP [6, 7]. In the largest multicenter study on MIDP, RDP was found to be associated with lower rates of conversion to open surgery, better spleen preservation and lower readmission rates despite a longer operative time and a prolonged hospital stay [6]. Although the robotic approach is a better approach for technically challenging cases, there is no difference in the incidence of postoperative complications.

Obesity is a risk factor for intraoperative difficulty in minimally invasive abdominal surgery, and prior studies have shown a higher rate of complications among obese patients undergoing pancreatectomy [8–11]. Robotic surgery in obese patients had better short-term outcomes than laparoscopy in selected patients undergoing visceral oncologic surgery [12, 13]. In a retrospective series describing the outcomes of approximately 3000 patients, the conversion rate of LDP was twice that of RDP (17.3% vs. 8.5%), and RDP was independently associated with a lower risk of unplanned conversion (OR 0.32; 95% CI 0.19–0.52) [14]. In the same study, patients with a higher body mass index (BMI), low preoperative albumin level, current smoking habit, and malignant T3/T4 disease or chronic pancreatitis had a higher risk of surgical conversion than those with benign tumors smaller than 5 cm [14].

Despite the major impact of a high BMI and visceral fat on MIDP-associated morbidity, there are no studies in the literature exclusively comparing RDP with LDP in obese patients. In this study, we hypothesize that the robotic approach is better for decreasing the risk of postoperative complications than LDP in obese patients. The primary aim of this study was to compare postoperative morbidity and short-term outcomes in RDP and LDP in patients with a BMI ≥ 30.

## Materials and methods

### Study design

This is a multicentric international study based on the retrospective analysis of a single database including all consecutive patients with obesity who underwent MIDP between January 2012 and January 2022 in 18 centers across 7 countries. A specific pseudoanonymized database was created by the coordinating center (Hospital Clinic, Barcelona, Spain) from different databases codified by each center with the variables of interest. The study was approved by the institutional review board of any participating center (coordinating center code: HCB/2022/0438) and conducted in accordance with the ethical principles of the Declaration of Helsinki.

Only centers performing both RDP and LDP were invited to participate in the study; also, participating centers must have performed at least 50 distal MIDP procedures and a minimum of 15 RDP procedures within the study period. Inclusion criteria were as follows: (a) patients aged > 18 years; (b) BMI ≥ 30; (c) MIDP performed for any indication. Exclusion criteria were: (a) initial open surgery; (b) MIDP in nonobese patients (BMI < 30) and (c) patients with a follow-up shorter than 90 days.

### Data collection

Demographic and clinical variables were analyzed. Patient demographics included age, sex and BMI. Other clinical factors included Charlson score, ASA classification, preoperative hemoglobin and albumin serum levels. Histories of previous abdominal surgery and medical comorbidities, including active smoking, preoperative diabetes, cardiovascular, respiratory, renal and hepatic disease, were also recorded. Preoperative details comprised anatomical and oncologic data and treatments, preoperative diagnosis and preoperative histology details, imaging tumor/lesion(s) features and diameter. Surgical characteristics, including the type and extent of pancreatic resection, indications for preoperative planning of spleen-preservation distal pancreatectomy (SPDP) and its type (with or without splenic vessel preservation), devices and materials used, surgical technique details and surgeon experience with robotic pancreatectomy, were recorded. The number of cases required to achieve proficiency and to be considered a surgeon experienced in performing RDP according to the learning curve was set at 20 procedures [15]. Data from the pathology report were also collected, such as final histology, size of lesions and number of lymph nodes retrieved in cases of malignancy.

### Preoperative evaluation and surgical management

All patients underwent preoperative abdominal computed tomography (CT) and pancreatic magnetic resonance imaging when indicated. Multidisciplinary teams personalized the best strategy for oncologic patients according to each center’s policy. The aim of surgical treatment is to achieve complete resection of the tumor with regional lymphadenectomy in cases of pancreatic ductal adenocarcinoma (PDAC) or other malignancies. In the case of benign disease (i.e., cystic lesions or chronic pancreatitis) or low-risk (pre)-malignant disease classified as well-differentiated pancreatic neuroendocrine tumor (PNET) smaller than 2 cm, low-risk intraductal papillary mucinous neoplasm (IPMN) or other lesions with any grade of dysplasia without evidence of malignancy, SPDP was considered according to each center program. Data on multivisceral en bloc resection, vascular resection (i.e., left renal vein), and radical antegrade modular pancreatosplenectomy (RAMPS) were collected.

### Clinical outcomes

The main outcomes included postoperative morbidity and mortality at 90 days, conversion to open surgery, intraoperative complications, operative time, estimated blood loss and data related to blood transfusion. Intraoperative complications were defined as any event necessitating treatment (i.e., bleeding requiring transfusion or lesion of an anatomical structure needing unplanned intervention) causing deviation from the ideal intraoperative course occurring between skin incision and skin closure. Postoperative complications were classified according to the Clavien‒Dindo (CD) classification [16], and severe morbidity was defined as CD grade ≥ 3. The Comprehensive Complications Index (CCI) was used to assess morbidity in patients with multiple complications [17]. The clinically relevant postoperative pancreatic fistula (POPF) rate and its grading according to the International Study Group on Pancreatic Surgery (ISGPS) definition [18], reoperation rate, delayed gastric emptying, postoperative hemorrhage and wound infection rate were also collected.

The length of hospital stay, time to regular diet, and readmission rate were also registered. When SPDP was planned, the proportion of spleen that was preserved and the reasons for unplanned splenectomy were registered. Conversion was defined by the need for a laparotomy to complete the laparoscopic operation in an instance of technical complexity or complications.

### Statistical analysis

Quantitative variables are described as medians and interquartile ranges (25th and 75th percentiles), and qualitative variables are described as absolute frequencies and percentages. To account for allocation bias, the inverse probability treatment weighting (IPTW) approach was calculated from the propensity score (PS) to predict the probability of assignment to the RDP group [19, 20]. This PS estimation was obtained from a logistic regression model based on variables that could be associated with treatment selection and other prognostically important covariates, including age, sex, BMI, Charlson and ASA scores, surgeon experience with robotic surgery, smoking habit, comorbidity, previous abdominal surgery, planned spleen preservation, preoperative histologic diagnosis, nature of lesions, involvement of other organs and diameter and location of tumor. The homogeneity of both groups (RDP and LDP) of baseline variables from the resultant pseudopopulation after applying IPTW was assessed using standardized differences (STD), i.e., differences divided by the pooled standard deviation (SD) between both groups. Proper balance of all matching covariates was pursued by using an after ± 0.20 cutoff point for STD [21].

Differences between groups by IPTW weighted logistic regression analysis were used to estimate odds ratio (OR) and their 95% confidence interval (95% CI) of developing a previous clinical outcome in case of dichotomous outcome or IPTW weighted Mann‒Whitney U test for quantitative outcomes. SPSS version 26.0 (IBM, Armonk, NY, USA) and SAS version 9.4 (SAS Institute, Cary, NC, USA) were used for data management and analysis.

## Results

### Demographics and perioperative characteristics

Overall, 436 patients underwent either a LDP (n = 217, 49.8%) or an RDP (n = 219, 50.2%). Table 1 shows the demographic and perioperative characteristics. Standardized differences of raw data on sex, smoking habit, preoperative albumin serum level, surgeon experience in RDP, preoperative histology and adjacent organ involvement exceeded the ± 0.20 cutoff point. However, after IPTW, the STD showed an appropriate balance of covariates between the RDP and LDP groups, so no inferential analysis was performed to compare the groups (Table 1). The overall median age was 60 (interquartile range {IQR} 49–70) years, and the median BMI was 33 (IQR 31–36). Approximately half of the patients (49.8%) previously underwent abdominal surgery and were classified as ASA III or higher (59.6%). The clinical diagnosis was benign in 26.1% of cases and premalignant in 48.2%. Preoperative biopsy identified PDAC in 21.6% and PNET in 20.9% of cases. The median diameter of pancreatic lesions was 28 mm, and the pancreatic tail was the most common location (54%). An SPDP was initially planned in approximately one-third of the entire population (29.8%) (Table 1).

**Table 1:** Robotic versus laparoscopic distal pancreatectomy in obese patients: demographics and preoperative characteristics

Variables	Robotic distal pancreatectomy (n = 219)	Laparoscopic distal pancreatectomy (n = 217)	Overall (n = 436)	Standardized difference (raw data)*	Standardized difference (After IPTW)
Age (years), median (IQR)	61 (49–70)	59 (49–70)	60 (49–70)	0.026	− 0.01
Female gender, n (%)	136 (62.1)	104 (47.9)	240 (55.1)	**0.288**	0.034
BMI (kg/m^2^), median (IQR)	32.8 (31–36)	33 (30.7–36)	33 (31–36)	− 0.065	− 0.02
Charlson score, median (IQR)	3 (2–6)	4 (2–6)	4 (2–6)	− 0.15	− 0.01
ASA status, n (%)					
I	3 (1.4)	11 (5.1)	14 (3.2)		
II	80 (36.5)	82 (37.8)	162 (37.2)		
III	131 (59.8)	114 (52.5)	245 (56.2)		
IV	5 (2.3)	10 (4.6)	15 (3.4)	0.093	− 0.04
ASA ≥ III, n (%)	136 (62.1)	124 (57.1)	260 (59.6)	0.101	− 0.07
Previous abdominal surgery, n (%)	108 (49.3)	109 (50.2)	217 (49.8)	− 0.018	0.098
Smoking habit, n (%)	61 (27.9)	35 (16.1)	96 (22)	**0.286**	− 0.173
Any comorbidity, n (%)	156 (71.2)	163 (75.1)	319 (73.2)	− 0.088	− 0.102
Cardiovascular disease, n (%)	61 (27.9)	96 (44.2)	157 (36)		
Pulmonary disease, n (%)	46 (21)	50 (23)	96 (22)		
Kidney disease, n (%)	22 (0.1)	17 (7.8)	39 (8.9)		
Hepatic disease, n (%)	17 (7.8)	13 (6)	30 (6.9)		
Preoperative diabetes, n (%)	51 (2.3)	59 (27.2)	110 (25.2)		
Comorbidity > 1, n (%)	98 (44.8)	89 (41)	187 (42.9)		
Hemoglobin serum level, g/dl, median (IQR)	13.1 (12.3–14.1)	13.4 (12.4–14.4)	13.3 (12.4–14.3)	− 0.127	0.002
Albumin serum level g/dl, median (IQR)	37 (34–42)	41 (34–43.3)	39 (29–43)	− **0.252**	− 0.08
Surgeon robotic experience > 20 procedures	176 (80.4)	118 (54.4)	294 (67.4)	**0.813**	0.114
Planned spleen preservation, n (%)	73 (33.3)	57 (26.3)	130 (29.8)	0.156	0.045
Lesion location imaging, n (%)					
Neck	11 (5)	8 (3.7)	19 (4.4)	0.163	0.12
Body	75 (34.3)	62 (28.6)	137 (31.4)		
Tail	111 (50.7)	128 (59)	239 (54.8)		
Diffuse (≥ 2 sites)	22 (10.1)	19 (8.7)	41 (9.4)		
Tumor type imaging, n (%)					
Benign	56 (25.6)	58 (26.7)	114 (26.1)		
(Pre)-Malignant	105 (47.9)	105 (48.4)	210 (48.2)		
PDAC	33 (15.1)	35 (16.1)	68 (15.6)		
Other	19 (8.7)	11 (5.1)	30 (6.9)	0.194	0.085
Unknown	6 (2.7)	8 (3.7)	14 (3.2)		
Preoperative histology, n (%)					
PDAC	65 (29.7)	29 (13.3)	94 (21.6)		
PNET	54 (24.7)	37 (17.1)	91 (20.9)		
Benign	19 (8.7)	9 (4.2)	28 (6.4)		
(Pre)-Malignant	24 (11)	21 (9.7)	45 (10.3)	**0.711**	0.146
Other	13 (6)	21 (9.7)	34 (7.8)		
No biopsy	44 (20)	100 (46)	144 (33)		
Preoperative adjacent organ involvement, n (%)	5 (2.3)	10 (4.6)	15 (3.4)	**0.218**	0.023
Tumor size imaging, mm, median (IQR)	29 (17–40)	28 (20–44)	28 (18–40)	− 0.136	0.032

*IPTW* inverse probability treatment weighting, *IQR* inter-quartile range, *BMI* body mass index, *ASA* American society of anesthesiologists, *IPMN* intraductal papillary mucinous neoplasm, *PNET* pancreatic neuroendocrine tumor, *PDAC* pancreatic ductal adenocarcinoma

*In bold preoperative standardized differences ± 0.20

Table 2 shows the surgical and technical details. In the majority of patients (57.1%), pancreas transection was performed at the pancreatic isthmus in 2/3 of the cases (72.7%) with a stapler device. The RAMPS technique was performed in 16.6% of patients with PDAC, and the en bloc adjacent organ resection rate was 4.6%. The most frequent indication for SPDP was cystic benign lesions (33.8%), while low-risk IPMN accounted for only 7.7%. The most frequent technique used was vessel preservation-SPDP (61.6%). The most frequent lesion on final pathology was low-grade PNET (30.7%); PDAC was finally diagnosed in 18.6% of patients and IPMN in 10.8% (Table 2).

**Table 2:** Robotic versus laparoscopic distal pancreatectomy in obese patients: perioperative characteristics

Variables	Robotic distal pancreatectomy (n = 219)	Laparoscopic distal pancreatectomy (n = 217)	Overall (n = 436)
Indication spleen preservation			
IPMN low risk	8 (11)	2 (3.5)	10 (7.7)
Serous/mucinous cystadenoma	27 (37)	17 (29.8)	44 (33.8)
PNET < 2 cm	19 (26)	20 (35.1)	39 (30)
Other	19 (26)	18 (31.6)	37 (28.5)
Pancreas texture, n (%)			
Soft	58 (26.5)	41 (18.9)	99 (22.7)
Hard	46 (21)	38 (17.5)	84 (19.3)
Unknown	115 (52.5)	138 (63.6)	253 (58)
Site of pancreas transection, n (%)			
Neck/isthmus	131 (59.8)	118 (54.4)	249 (57.1)
Body (further to the left)	88 (40.2)	99 (45.6)	187 (42.9)
Stump closure method, n (%)			
Stapler	151 (68.9)	166 (76.5)	317 (72.7)
Suture	30 (13.7)	29 (13.4)	59 (13.5)
Ultrasonic device	19 (8.7)	7 (3.2)	26 (6)
Other	12 (5.5)	5 (2.3)	17 (3.9)
Unknown	7 (3.2)	10 (4.6)	17 (3.9)
RAMPS, n (%)	18 (8.2)	54 (24.9)	72 (16.5)
Type of spleen preservation, n (%)			
Nimura	28 (59.6)	25 (64.1)	53 (61.6)
Warshaw	4 (8.5)	14 (35.9)	18 (20.9)
Unknown	15 (31.9)	0	15 (17.5)
Vascular resection n (%)			
SMV/PV	1 (0.46)	1 (0.46)	2 (0.46)
Left renal vein	1 (0.46)	2 (0.9)	3 (0.7)
Adjacent organs resected, n (%)	9 (4.1)	11 (5.1)	20 (4.6)
Wound incision extraction site			
Pfannestiel	73 (33.3)	40 (18.4)	113 (25.9)
Midline	62 (28.3)	81 (37.3)	143 (32.8)
Other	84 (38.4)	96 (44.3)	180 (41.3)
No. of lymph nodes retrieved, median (IQR)	18 (11–23)	13 (7–21)	16 (9–23)
Final histology, n (%)			
PNET G1/G2	70 (32)	64 (29.5)	134 (30.7)
PNET G3	3 (1.4)	4 (1.8)	7 (1.6)
PDAC	37 (16.9)	44 (20.3)	81 (18.6)
Mucinous cystic neoplasm	21 (9.6)	22 (10.1)	43 (9.9)
Serous cystadenoma	20 (9.2)	7 (3.2)	27 (6.2)
IPMN	29 (13.2)	18 (8.3)	47 (10.8)
Solid pseudopapillary tumor	10 (4.6)	13 (6)	23 (5.3)
Chronic pancreatitis/pseudocyst	8 (3.4)	20 (9.2)	28 (6.4)
Metastasis	7 (3.2)	6 (2.8)	13 (2.9)
Other	12 (5.6)	19 (8.8)	31 (7.1)
Unknown	2 (0.9)	–	2 (0.5)

*IPMN* intraductal papillary mucinous neoplasm, *PNET* pancreatic neuroendocrine tumor, *G 1–3* grade, *PDAC* pancreatic ductal adenocarcinoma, *RAMPS* radical antegrade modular pancreatosplenectomy, *SMV/PV* superior mesenteric vein/portal vein, *IQR* inter-quartile range

### Postoperative morbidity and operative outcomes

The outcomes after the IPTW comparison method are shown in Table 3. The overall complication rate was 57.8%. Overall, 21% of POPF cases were clinically relevant, the reoperation rate was 3.9% and the rate of postoperative hemorrhage was 2.3%. The risk of suffering any postoperative complication was significantly lower with the robotic approach (OR 0.68, 95% CI 0.52–0.89; p = 0.005), as was the risk of suffering multiple complications (OR 0.54, 95% CI 0.40–0.74; p < 0.001 (Table 3). Therefore, the median CCI was also significantly lower in the RDP group than in the LDP group: 8.7 (8.7–8.9) vs. 8.9 (8.9–15), respectively (p < 0.001). In contrast, the severity of complications according to the CD classification as well the occurrence of clinically relevant POPF and the reoperation rate were comparable in both groups. The wound infection rate and the incidences of delayed gastric emptying and postoperative hemorrhage were also similar between the RDP and LPD groups (Table 3).

**Table 3:** Robotic versus laparoscopic distal pancreatectomy in obese patients: operative and short-term outcomes after IPTW matching

Variables	Robotic distal pancreatectomy (n = 219)	Laparoscopic distal pancreatectomy (n = 217)	Odds ratio (95% Confidence interval)^$^	p value^$^
CCI, median (IQR)	8.7 (8.7–8.9)	8.9 (8.9–15.0)		** < 0.001**
Patients with any postoperative complication, n (%)	123 (56.2)	129 (59.5)	0.68 (0.52–0.89)	**0.005**
Patients with postoperative complications > 1, n (%)	52 (23.7)	57 (26.3)	0.54 (0.40–0.74)	** < 0.001**
CD classification, n (%)				
I / II	142 (74)	154 (73.7)		
III a/b	41 (21.3)	45 (21.5)	0.76 (0.53–1.08)	0.125
IV a/b	8 (4.2)	10 (4.8)		
V	1 (0.5)	–		
Conversion to open, n (%)	28 (12.8)	59 (27.2)	0.27 (0.19–0.39)	** < 0.001**
Intraoperative complications, n (%)	6 (2.7)	17 (7.9)	0.17 (0.08–0.35)	** < 0.001**
Operative time (min), median (IQR)	272 (220–358)	270 (209–325)		0.249
Blood loss (ml), median (IQR)	150 (80–250)	200 (100–500)		** < 0.001**
Intraoperative blood transfusion, n (%)	6 (3.9)	18 (10.6)	0.28 (0.15–0.50)	** < 0.001**
No RBC units transfused, median (IQR)	2 (2–4)	2 (2–4)		**0.015**
Spleen preservation intended and finally preserved *	47/73 (64.4)	39/57 (68.4)	1.96 (1.13–3.39)	**0.016**
Reoperation, n (%)	10 (4.6)	7 (3.2)	1.42 (0.63–3.18)	0.400
POPF clinically relevant, n (%)	38 (17.4)	53 (24.5)		
B1	15 (39.5)	31 (58.5)	0.78 (0.56–1.10)	0.157
B2	15 (39.5)	9 (17)		
B3	4 (10.5)	10 (18.9)		
C	4 (10.5)	3 (5.6)		
Postoperative hemorrhage, n (%)	4 (1.8%)	6 (2.8%)	0.85 (0.32–2.25)	0.745
Wound Infection, n (%)	6.9%	7.4%	1.28 (0.76–2.17)	0.354
Delayed gastric empting, n (%)	21 (9.7%)	32 (14.8%)	0.70 (0.46–1.05)	0.082
Time to regular diet, days, median (IQR)	3 (3–4)	4 (4–5)		** < 0.001**
Hospital stay, days, median (IQR)	6 (6–7)	6 (6–7)		0.311
Readmission, n (%)	47 (21.5)	48 (21.1)	0.74 (0.54–1.02)	0.066

*IPTW* inverse probability treatment weighting, *IQR* interquartile range, *OR* odds ratio, *CCI* comprehensive complication index, *CD* Clavien–Dindo, *POPF* post-operative pancreatic fistula

*Calculated only in case of planned spleen preservation

^$^Statistical inferential analyses performed with IPTW calculation as weight for logistic regression analysis or Mann–Whitney U test. Bold values indicate a statistically significant difference. Results are described by absolute frequencies and percentage (raw data) for qualitative variables and estimated median and 95% Confidence interval from ITPW analysis for quantitative variables

The operative time did not differ between the groups. The overall conversion rate was 19.9%. After IPTW, RDP was independently associated with a lower conversion rate than LDP, with an OR of 0.27 (95% CI 0.19–0.39; p < 0.001). RDP was not a risk factor for intraoperative complications after IPTW (OR 0.17, 95% CI 0.08–0.35; p < 0.001). In 19 out of 23 patients who had a significant intraoperative adverse event, an emergency conversion to open surgery was needed to control bleeding. Median blood loss was significantly higher in the LDP group than in the RDP group (200 ml vs. 150 ml; p < 0.001), as was the intraoperative need for transfusion (OR 0.28, 95% CI 0.15–0.50; p < 0.001). Patients who underwent RDP had less red blood cell units transfused (p = 0.015) (Table 2). The postoperative need for transfusion was comparable between both groups (OR 0.75, 95% CI 0.45–1.23; p = 0.254). When SPDP was initially planned, the success of the robotic approach was twofold higher than that of the LDP (OR 1.96, 95% CI 1.13–3.39). Length of hospital stay was not significantly different between the RDP and LDP groups (6 days each); however, the time needed to achieve a regular diet was significantly lower in the RDP group (3 vs. 4 days; p < 0.001). The readmission rate was also similar (21% in each group) regardless of the approach used (OR 0.74, 95% CI0.54–1.02; p = 0.066).

### Mortality

Mortality at 90 days was 0.7% (3 patients). A patient in the RDP group died of lung complications after POPF (CD grade V, see Table 3). Another patient in the RDP group died of unknown causes (no intra- or postoperative complications). A patient in the LDP group died of early progression of PDAC.

## Discussion

This is the first study demonstrating that RDP in obese patients is superior to LDP not only in conversion rate, blood loss and spleen preservation but also in terms of postoperative complications.

MIDP has better short-term outcomes than open surgery. Two randomized controlled trials (The LEOPARD-1 trial from the Netherlands and the LAPOP trial from Sweden) included patients with benign and premalignant disease and reported a shorter time until functional recovery, shorter length of hospital stay and comparable postoperative morbidity [1, 22]. Despite the introduction of robotics for pancreas surgery almost 20 years ago [23] and the lack of randomized controlled trials (RCTs) comparing RDP and LPD or ODP, there is growing evidence within the surgical community that the robotic approach is superior to any other approach [24]. However, the use of a minimally invasive approach for malignant left-sided tumors is still under debate, and data from RCTs remain unpublished.

Although it is complex to design studies that can confirm the superiority of the robotic approach, most surgeons believe that the robotic approach even allows MIDP to be performed in technically complex cases. Evidence supporting the superiority of RDP over LPD comes from retrospective studies showing that RDP has a shorter learning curve, lower conversion rates to open surgery and causes less blood loss [6, 25, 26]. Despite these advantages, there is no evidence that the complication rate is significantly different between the techniques. However, it is unlikely that RCTs comparing RDP and LDP will be performed in the future since many institutions have already began performing MIDP using a robotic approach rather than using a laparoscopic approach. For this reason, we decided to investigate whether the robotic approach could make a significant difference in difficult technical conditions and in patients with a higher risk of complications.

Obesity has dramatically increased over the last four decades in Western countries, and the prevalence in the USA and UK has reached up to 35% in the last decade [27]. Patients with a very high BMI undergoing minimally invasive surgery have poorer visualization of the surgical field due to adipose tissue accumulations in mesenteries (bowel, omentum, pelvic peritoneum), and sometimes insufficient length of instruments, perceived stiffness of the abdominal wall due to the fulcrum effect and uncomfortable position can make it difficult to control an emergency and increase the risk of injury during surgery. Additionally, obese patients have a much greater risk of potentially life-threatening complications after undergoing surgery, such as cardiovascular events, wound and urinary infections, and venous thromboembolism [28]. In fact, the obese patients in our manuscript who underwent the robotic approach had poor postoperative outcomes poorer according to the reported median benchmark values for nonobese patients [5]. The conversion rate can reach up to 12.8% (four times higher than the benchmark cutoff), the operation time is longer (239 vs. 272 min), the CCI and number of patients with severe (CD > 2) complications (17.8% vs. 13.3%) are higher, and the risks of pancreatic fistula development (17.4% vs. 13.3%) and readmission (21.5% vs. 11.7%) are higher.

In our study, we hypothesized that a robotic approach in obese patients could overcome some technical difficulties associated with laparoscopy and therefore could significantly improve perioperative outcomes and, most importantly, reduce the incidence of postoperative complications. Most short-term outcomes of RDP were significantly better than those of LDP, demonstrating that RDP was clearly beneficial for patients. The reason for these differences might be that the robotic approach was less likely to require conversion to open surgery. Conversion to open surgery is not (and should not be) considered a complication, as it is representative of good surgical judgment if a problem occurs intraoperatively. However, surgery is often associated with intraoperative difficulties such as poor exposure of the surgical field, intraoperative bleeding (emergency conversion) and injury to other organs. These events are associated with poorer postoperative outcomes, and laparotomy itself can hinder postoperative mobilization of the patient and increase wound infections [10].

The main limitation of this study is its retrospective nature. However, the data were collected from high-volume centers with prospectively managed databases. Additionally, the use of the IPTW approach mitigated the biases of group allocation and selection of surgical approach. Second, we did not analyze the differences in the oncologic results between the groups; however, we believe this analysis goes beyond the purpose of this study, and a different group of patients would need to be selected to investigate these outcomes. Finally, we did not consider the potential impact of a specific learning curve for obese patients; however, we only included data from expert centers, and we assessed the experience of surgeons to avoid the bias of lack of experience in RDP.

In obese patients, RDP is superior to LDP in terms of postoperative complications and most short-term outcomes; therefore, it should be considered the best approach in cases of benign and premalignant disease. Further studies are needed to confirm the oncological outcome in obese patients with malignant disease.
